# Multimaterial Printing of Liquid Crystal Elastomers with Integrated Stretchable Electronics

**DOI:** 10.1021/acsami.2c23028

**Published:** 2023-05-10

**Authors:** Michael R. Vinciguerra, Dinesh K. Patel, Wuzhou Zu, Mahmoud Tavakoli, Carmel Majidi, Lining Yao

**Affiliations:** † Department of Mechanical Engineering, Carnegie Mellon University, 5000 Forbes Ave., Pittsburgh, Pennsylvania 15213, United States; ‡ Human Computer Interaction Institute, Carnegie Mellon University, 5000 Forbes Ave., Pittsburgh, Pennsylvania 15213, United States; § Institute of Systems and Robotics, Department of Electrical Engineering, University of Coimbra, Coimbra 3090-290, Portugal

**Keywords:** digital fabrication, 3D printing, 4D printing, liquid crystal elastomer (LCE), liquid metal, soft robotics

## Abstract

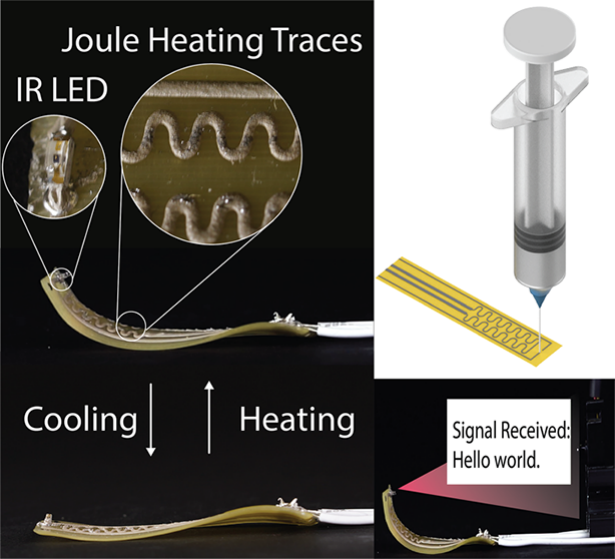

Liquid crystal elastomers (LCEs) have grown in popularity in recent years as a stimuli-responsive material for soft actuators and shape reconfigurable structures. To make these material systems electrically responsive, they must be integrated with soft conductive materials that match the compliance and deformability of the LCE. This study introduces a design and manufacturing methodology for combining direct ink write (DIW) 3D printing of soft, stretchable conductive inks with DIW-based “4D printing” of LCE to create fully integrated, electrically responsive, shape programmable matter. The conductive ink is composed of a soft thermoplastic elastomer, a liquid metal alloy (eutectic gallium indium, EGaIn), and silver flakes, exhibiting both high stretchability and conductivity (order of 10^5^ S m^–1^). Empirical tuning of the LCE printing parameters gives rise to a smooth surface (<10 μm) for patterning the conductive ink with controlled trace dimensions. This multimaterial printing method is used to create shape reconfigurable LCE devices with on-demand circuit patterning that could otherwise not be easily fabricated through traditional means, such as an LCE bending actuator able to blink a Morse code signal and an LCE crawler with an on/off photoresistor controller. In contrast to existing fabrication methodologies, the inclusion of the conductive ink allows for stable power delivery to surface mount devices and Joule heating traces in a highly dynamic LCE system. This digital fabrication approach can be leveraged to push LCE actuators closer to becoming functional devices, such as shape programmable antennas and actuators with integrated sensing.

## Introduction

Liquid crystal elastomers (LCEs) are a class of materials that combine the ordered direction of liquid crystals with the properties of elastomers and exhibit shape memory properties.^1−7^ These materials are formed from mesogens and acrylate terminated chains that when heated above the nematic-to-isotropic transition temperature (*T*_NI_) undergo macroscopic contraction along the direction of the mesogens. Upon cooling, these materials relax back to their original state, exhibiting reversible actuation. Because of this material property and their intrinsic softness, these materials have been explored in recent years for use in wearable devices,^8,9^ soft robotics,^10,11^ and other shape-changing architectures.^12^ In more recent work, LCE has been patterned using additive manufacturing techniques like 3D printing, expanding the range of shapes that can be achieved with these materials, known as “4D printing”.^13−20^ Ultraviolet (UV) light is used during the printing process to initiate the photopolymerization of the LCE, locking the liquid crystal mesogens and chains into place and aligning individual LCE fibers with respect to the printing direction. As a result, the resolution of control over the direction of contraction is limited only by the diameter of the printing nozzle and the 3D printer resolution. By layering LCE in different configurations using this digital fabrication approach, out-of-plane bending and other motions dictated by the orientation of the monodomain material can be achieved from initially flat sheets.^21^

Despite these advances in fabricating LCEs, researchers have only just started looking into how to provide additional functionality beyond temperature- or light-controlled actuation.^22,23^ To be useful for soft robotic systems, LCEs must incorporate computation, sensing, and directly controllable actuation into a single architecture. Accomplishing all of these goals simultaneously can be accomplished by introducing electrical elements into the LCE. While LCEs are not intrinsically conductive, work has been done recently to introduce wiring or metal inclusions to power LCE actuators. One element that has been previously studied is the use of Joule resistive heating to provide heat to LCEs to actuate them. Past work has used serpentine traces of metal,^24−26^ carbon nanoparticle,^27^ silver ink,^18,28,29^ and liquid metal^30−32^ as conductive traces that are embedded within the LCE or adhered to the surface. However, each of these methods has its limitations. Serpentine metal traces are flexible and stretchable in certain directions but introduce highly localized stiffness mismatches that can lead to delamination during contraction. Nanoparticle composites, including carbon-filled LCEs, require large weight fractions of filler material to become conductive, leading to increased stiffness or mechanical hysteresis that can interfere with the reversibility and actuation stroke of LCEs. Liquid metal (LM) alloys, like eutectic gallium–indium (EGaIn), have attractive qualities that address some of these issues; they are highly conductive^33^ and intrinsically soft, thereby able to deform with the surrounding LCE with no degradation over many actuation cycles. LM can be incorporated directly into the LCE in the form of a percolating network of microscopic inclusions,^30,32,34^ as microfluidic channels^31,35^ or through coaxial printing methods.^36^ These approaches provide high conductivity to LCEs while minimally impacting their mechanical compliance or ability to contract during electrical stimulation and have been shown to work well for closed-loop feedback control.^36^ LM channels have also been used previously to provide proprioception of robotic actuators.^37^ Despite these advances, introduction of electronic components such as surface mount devices or integrated circuits into or on the LCE has not yet been demonstrated, limiting the scope and applicability of these structures. LM by itself serves as a poor candidate for the integration of electronics because it can smear or leak and cannot be used to sinter electrical devices to the LCE.

To improve the ability to incorporate complex LM-based electronics into soft material systems, researchers have recently introduced a new class of highly conductive and stretchable inks composed of EGaIn, Ag flakes, and a polystyrene–isoprene–styrene (SIS) hyperelastic binder that can be printed using direct ink writing (DIW).^38,39^ Because the material is initially diluted in a solvent, it exhibits shear thinning behavior needed for DIW-based deposition. Once the solvent evaporates, the conductive ink displays a low electromechanical coupling, exhibiting only a small change in its initial resistance over many loading cycles as compared to resistive silver inks and other materials that can degrade or delaminate.^38,39^ The material can strain >100% in many cases with little change to its resistance. The average conductivity of the EGaIn-Ag-SIS ink from prior studies^38,39^ is on the order of 10^5^ S m^–1^; the trade-off between the ink’s conductivity when compared to that of its constituent materials (about 3.4 × 10^6^ S m^–1^ for EGaIn,^40^ 6.3 × 10^7^ S m^–1^ for silver^41^) comes with the added benefit of the SIS matrix that allows the ink to bond well to various surfaces. All of these factors contribute to the ability to integrate the conductive ink with the printed LCE substrates. LCEs operate within the range of 10–40% actuation strain,^1^ a range in which this conductive ink will see very little change to its resistance over hundreds of cycles, in contrast to other conductive inks. Additionally, the SIS matrix will allow the conductive ink to stay attached to the surface of the LCE and prevent delamination.

**Figure 1:**
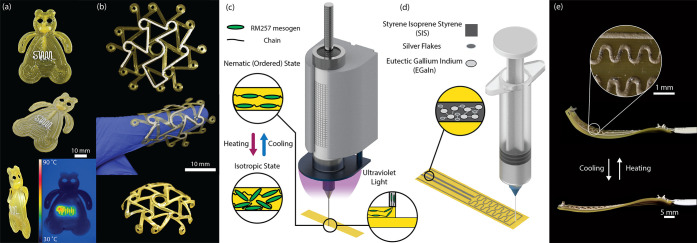
Overview of the multimaterial printing process. (a) 4D printed liquid crystal elastomer (LCE) teddy bear with integrated Joule heating trace that was digitally fabricated using the approach outlined in this work. (b) A chiral auxetic-like structure with arbitrarily patterned conductive ink. The actuated state is shown at the bottom of the figure with the conductive ink on the inner side of the actuator. (c) 4D printing of the LCE. As the ink leaves the nozzle, the mesogens and chains are aligned along the printing direction, and the direction of contraction is locked using the ultraviolet (UV) light array. (d) 3D printing of EGaIn-Ag-SIS ink. The conductive ink is patterned directly on top of an LCE substrate. (e) An LCE bending actuator with an infrared LED. Bending is induced by creating a multilayer structure with layers at 0°–90° configurations. The conductive ink can contract with the LCE, providing consistent heating and power to ICs.

In this work, we introduce a novel digital fabrication methodology in which the EGaIn-Ag-SIS ink and LCE are printed to create shape reconfigurable 4D structures using a common DIW fabrication approach (Figure 1). This multimaterial printing approach is composed of several key elements that stand out from other efforts to print conductive inks and LCE. First, the LCE flow rate parameters are empirically tuned to produce a smooth surface for the conductive ink to be patterned onto. Next, we show that adding a final layer of LCE with the UV light turned off is useful for improving the final surface roughness (<10 μm deviation from average surface height) of the LCE substrates. Once the LCE samples are cured with UV light and the EGaIn-Ag-SIS ink is patterned on top, the conductive ink can potentially function as both a circuit wire for surface mount devices (SMDs) and other electrical components or as a Joule heating element. The repeatability of this printing process, with respect to the trace dimensions and conductivity, is demonstrated for 100+ printing traces. The conductive ink also allows for the transmission of heat up to the desired transition temperatures without a large change in the resistance of the traces. A linear relationship between normalized input power and the achievable Joule heating temperatures is also measured to provide designers with reference values for how these structures should be heated to achieve actuation.

To demonstrate the versatility of this multimaterial printing approach, we show for the first time the ability to combine LCE and EGaIn-Ag-SIS conductive ink to create LCE devices capable of simultaneous actuator and digital circuit functionality in a variety of form factors. For example, the teddy bear with conductive circuitry and chiral auxetic-like^42^ structures in Figure 1 would be difficult to create through conventional LCE fabrication methods. In addition to serving as general purpose, soft and stretchable circuitry, the LM-based ink can be used to provide localized heating of the structure that can be observed through infrared (IR) cameras. In particular, we demonstrate combining LCE Joule heating actuation and SMDs for use in a reconfigurable IR communication device and crawling robot. Other demonstrations with various SMDs such as LEDs and capacitive sensing are also demonstrated. With these advances, complex and functional LCE devices that push the boundary of what can be currently achieved with the material can be fabricated for use in applications like reconfigurable communications, soft robotics, and self-folding origami.

## Results

This methodology was evaluated on several different criteria: the ability to make smooth LCE surfaces to enhance the adhesion and printability of the conductive ink, the repeatability of conductive trace dimensions for specific flow rate parameters, the behavior of these traces as Joule resistive heaters, and applications of the overall printing methodology.

### Ink Synthesis, Characterization, and Printing

The LCE ink was synthesized using a one-step Michael addition reaction by combining RM-257 and *n*-butylamine in a 1:1.1 ratio along with a photoinitiator, a process adapted from a previous study.^17^ To determine what temperature the LCE needs to be Joule heated to in order to achieve full contraction, differential scanning calorimetry (DSC) is performed to find the characteristic *T*_NI_. The transition temperature of the material is around 80–90 °C (Figure S1).

The EGaIn-Ag-SIS ink is obtained by mixing SIS-block copolymer solution, silver flakes, and liquid metal in a ratio of 1:1.24:3 by weight. Electromechanical characterization was performed in the previous works^38,39^ and will not be focused on in this study.

Rheological characterization of each ink to determine their suitability for DIW 3D printing was performed. Frequency sweeps from angular frequencies of 10^–1^ to 10^2^ rad s^–1^ and viscosity measurements from shear rates of 10^–1^ to 10^1^ s^–1^ were obtained for each ink (Figure S2). The frequency sweeps for both materials (Figure S2a,c) suggest that they both behave more liquid-like under printing conditions due to the larger loss modulus *G*′′. In Figure S2b, the conductive ink is shown to be shear thinning, as the viscosity decreases with increasing shear rate. The Hyrel SDS-10 extruder used in this work is capable of extruding materials under a viscosity of 10^5^ cP^43^—therefore, the ink should be easily printable for a variety of nozzles. The largest limiting factor of the conductive ink’s printability is the early formation of AgIn_2_ solid deposits within the ink, which can physically clog the printing needles. To avoid this, it is recommended to mix each metal into the SIS block copolymer solution individually instead of adding both at the same time and then mixing the solution. In Figure S2d, the LCE ink is not shear thinning until potentially higher shear rates; however, at the printing temperature of 65 °C, the viscosity of the LCE ink is far under the Hyrel KRA extruder head’s viscosity limit of 10^6^ cP.^43^

After synthesizing the individual materials, they were combined in a multimaterial printing process (Figure 1). In Figure 1b, the LCE ink is loaded into a metal reservoir and printed using a heated extruder. The extruder must be hot enough to decrease the viscosity of the ink while not causing the material to transition to the isotropic state.^17^ As the ink leaves the nozzle, the mesogens and chains are aligned along the printing direction due to the shear stresses developed between the nozzle and the substrate. The orientation of the mesogens is locked into place using the mounted ultraviolet (UV) light array. A time lapse of the DIW LCE printing process is provided in Movie S1. The sample is then taken to cure in UV light for 10 h. Using the same printer, but a different extruder (Figure 1c), the conductive ink is patterned directly on top of the cured LCE substrate. The DIW printing process showing the deposition of the conductive ink onto an LCE surface is shown in Movie S2. The ink creates stretchable circuitry that can deform with the underlying LCE and provide both Joule heating and electrical power for integrated circuits (ICs) or SMDs (Figure 1a,c).

### LCE Surface Planarization

**Figure 2:**
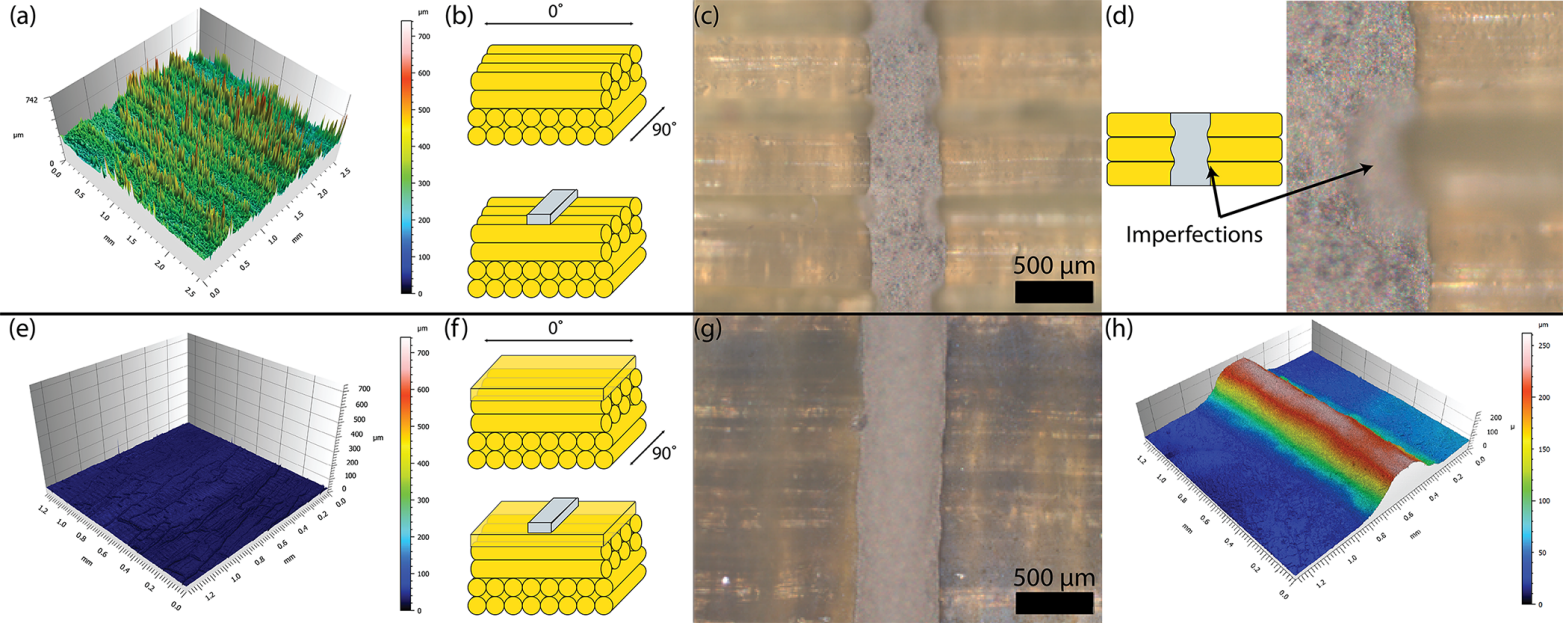
Confocal microscopy of printed LCE surfaces with conductive ink. (a) A 3D surface reconstruction of printed LCE with empirically tuned parameters. (b) Layer by layer construction of the composite structure. (c) Confocal microscopy image displaying the imperfections resulting from printing. (d) Zoomed-in confocal image to highlight the defects in the conductive ink. (e) A 3D surface reconstruction with the additional layer of uncured LCE, showing less than 10 μm deviation across the sample area. (f) Layer by layer construction of the composite structure with an additional layer of initially uncured LCE. (g) Confocal image showing the more uniform surface—there are less likely to be defects in the conductive ink traces. (h) A 3D surface reconstruction showing that the conductive traces are all consistent width throughout their length.

One of the primary concerns with multimaterial printing approaches revolves around providing a smooth interface between the different constituent materials (Figure 2). To achieve a smooth finish on the surface of the LCE actuators, the material flow rate and slicing parameters had to be empirically tuned to produce high-resolution prints. The surface of an LCE actuator using the empirically tuned parameters with and without conductive ink is shown in Figure 2a–d. While the surface is mostly uniform in height, there are small defects that can occur during printing. These deviations that occur at the interface of each line may prohibit conductive ink printing by either obstructing the nozzle, which prevents material deposition, or by creating gaps that cause the distance between the nozzle and substrate to increase, decreasing the strength of the shear thinning effect used to promote material deposition in DIW processes. In Figure 2c,d, a confocal microscopy image captures this phenomenon; due to irregularities in the LCE surface, the conductive ink is not patterned in a line of consistent width. Inconsistencies in the conductive ink create pockets of higher resistance on the devices, which can lead to improper forward voltages/currents for powering SMDs and/or uneven heating and burnout for Joule heating traces.

During normal printing operations, there can be a buildup of small defects due to the rheology of the ink, geometry processing, and the immediate photopolymerization of the LCE. Changing slicing parameters was found to reduce these issues but not eliminate them. To improve the surface finish, an extra layer of LCE is printed on top of the structure during printing. This LCE layer is not subjected to UV light, delaying the photopolymerization. Because of this, the material can flow as a very viscous liquid for a short amount of time and help fill in any gaps/cracks that may have formed from defects in the previous layers. The results of this additional fabrication step can be seen in Figure 2e–h. The surface topography of a characteristic sample of LCE with an extra layer of uncured material demonstrates only small (<10 μm) deviations in height. No noticeable defects in the conductive ink profile are observed when the sample is viewed under the confocal microscope (Figure 2g,h). Therefore, this “planarization” layer improves the printability of the conductive ink. In fabricating any actuator with the conductive ink, it is therefore recommended to print one extra layer with the UV light turned off to correct for any issues that can occur during printing. Alternatively, one can use the reverse side of the printed LCE, which has an intrinsic flatness due to its deposition on a flat printing bed. Additional surface analyses across samples printed without empirical tuning, with empirical tuning, and with empirical tuning plus the extra layer of LCE are given in the Supporting Information (Figure S3).

One concern that the introduction of this layer introduces is that it may remain polydomain or be unaligned, resulting in a decrease in the performance of any actuators using this approach. To determine the impact of this additional planarization layer, an experiment was conducted with linear LCE actuators where their contraction ratios were measured. The results of this experiment are demonstrated in Figure S4. LCE actuators were printed with several different layers to determine if the thickness of the actuator played a role in diminishing any effect that this extra layer might cause on the mechanical performance. For example, for a “single” layer structure, samples with a single layer of LCE printed with the UV light on are compared to two-layer LCE samples where a second “planarization” layer is printed without the UV light. This pattern continues for the other groups explored. The null hypothesis is constructed to assume that the average contraction of each type of sample would be the same, regardless of the use of UV light for the final layer. The alternative hypothesis is that the average contraction ratio of samples with the extra planarization layer will be smaller than the average contraction ratio of samples without the extra planarization layer. Before conducting the experiment, a power level of 0.01 was taken to determine if there was a significant difference between the samples. A student’s two sample *t* test was used to evaluate for significance. The probabilities are displayed in Figure S4. Because none of the probabilities are lower than the predefined power, we keep the null hypothesis and reject the alternative hypothesis. Therefore, there is no significant difference in contractile performance between samples with or without the extra planarization layer, and the effect of the planarization layer on actuation performance can be considered to be minimal.

To understand the phenomenon seen in Figure S4, polarized optical microscopy (POM) was conducted to determine the orientation of the mesogens in LCE actuators created using this printing process. The results of this analysis are shown in Figure S5. In the figure, the confocal images with the white light demonstrate that it is difficult to image the samples without polarized light because it is difficult to see the individual printed lines. In order to demonstrate that the mesogens are properly aligned within each sample, polarized light is introduced. If the mesogens are aligned, then the polarized light will be reflected back from the sample due to birefringence, except from areas between the individual traces, allowing us to better see these individual traces. In Figure S5a, a sample without the planarization layer is shown. The two layers are visible under polarized light but not under the normal light field, suggesting that the mesogens are properly aligned. In Figure S5b, a sample with the planarization layer is shown. The two layers are still visible under polarized light, albeit slightly dimmer than the two-layer structure with the UV light left on for both layers. This suggests that the mesogens are still aligned, albeit the layer might contain more unaligned mesogens than a normal sample. This, in addition to the relative thicknesses of 3D printed LCE when compared to thin films,^44^ likely explains the observations in Figure S4; the material can still contract, and even if the contraction ratio is smaller, the thickness of the actuators prevents the less active layers from affecting the rest of the structure.

### Repeatability of Manufacturing Process

**Figure 3:**
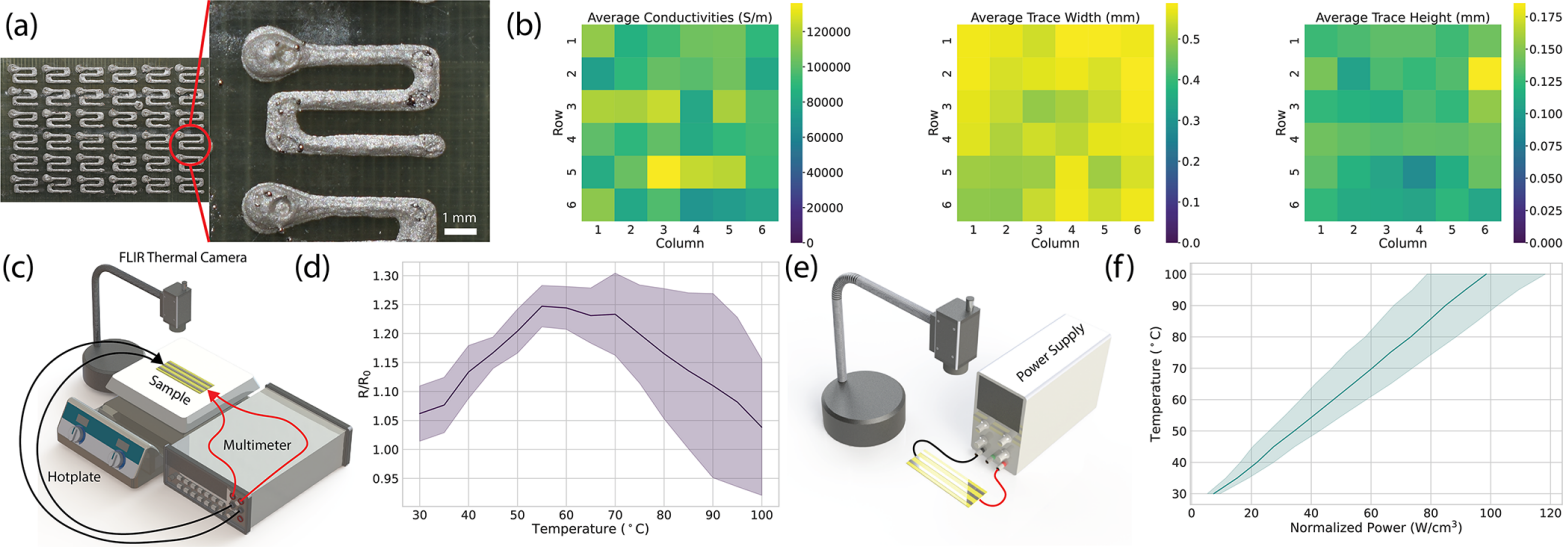
Characterization of the conductive ink on LCE substrates. (a) Example prints for repeatability tests. (b) Results of repeatability tests. (c) Experimental setup for measuring resistance as a function of temperature using a thermal camera, hot plate, and multimeter. (d) Change in resistance of *n* = 9 conductive traces as a function of temperature. The line represents the average values, and the shaded region represents the standard deviations. (e) Experimental setup for measuring temperature as a function of normalized input power. (f) Achievable temperatures with linear relationship on power input.

To demonstrate the repeatability of the conductive ink printing process, 108 conductive traces were printed on an LCE substrate. A single print yielded 107/108 (99.1%) electrically conductive lines. The dimensions of each line were measured using a confocal microscope, and the resistance of each line was measured using a 4-wire resistance measurement. Figure 3 shows that the dimensions are repeatable for a 27G nozzle. Figure 3a shows an example of the printed traces on an LCE substrate. The inset shows the three horizontal lines that were measured as part of each grouping to determine the average conductivities, widths, and heights. Figure 3b shows heatmaps characterizing the uniformity of the three properties measured. While the properties are mostly uniform, there are deviations in the heatmaps that we will address here. The trace dimensions measured were used to calculate the bulk electrical conductivities by assuming a rectangular cross section and applying Pouillet’s law. With regards to the conductivities, the average value across all of the samples was on the order of 10^5^ S m^–1^, but certain samples had slightly higher and lower conductivities. This is likely due to small variations in the rectangular cross section along the length of the wire that could not be accurately captured by just measuring the trace widths and heights at the horizontal center of each trace. Furthermore, slight deviations with each of these parameters could have resulted from a slightly nonuniform printing bed. While the LCE substrate is smooth, the bed can deviate in places up to 100 μm, which would have an impact on all of the measured and derived quantities. A sample without the planarization layer was also constructed (Figure S6), but confocal microscopy revealed that the cross-sectional area of the conductive traces were too inconsistent to perform substantial characterization.

### Thermal Behavior of Conductive Ink

For these composite devices to function as intended, the conductive ink must retain its electrical functionality after being applied to an LCE substrate. Because the ink is composed of a thermoplastic binder that melts at higher temperatures, the behavior of this ink up to and around the transition temperature of the LCE was studied. Three LCE substrates were manufactured with three conductive traces each for a total of nine samples.

Figure 3c shows the experimental setup to determine how the resistance of the traces change as a function of temperature. A FLIR thermal camera was used to measure the temperature of conductive ink traces printed on LCE actuators as the composite was heated by a hot plate. Once a trace reached a desired temperature, a probe resistance measurement was taken. The change in the resistance divided by the initial resistance of each trace at the end of three heating cycles is exhibited in Figure 3d. The results were captured after three heating cycles to reduce any Mullins effect that may have occurred as the material was heated and potentially stretched by the underlying LCE substrate. The resistance of each trace increases slightly initially before receding closer to the original values once the samples reach *T*_NI_. These results suggest that the circuitry will still perform well for a variety of purposes on LCE structures, including as digital circuitry—for example, the largest change in initial resistance between the first cycle of actuation and the start of the third cycle was observed to be 0.984 Ω, changing from 1.708 to 0.724 Ω. These values are still well within the range of viability for digital circuitry. Because the resistance changes are very small despite the large temperature changes, designers will not have to worry about large changes in input power. Therefore, SMDs that rely on stable forward voltages/currents can continue to operate while the LCE substrate is actuated.

An experiment using the same nine samples was conducted to determine what power input was required to achieve specific temperatures (Figure 3e). Because one of the proposed use cases for the ink was Joule heating to drive the actuation of the LCEs, it is important to determine what temperatures are achievable by the conductive ink. In these experiments, each trace was connected to a voltage-controlled power supply and allowed to Joule heat for 1 min to establish a quasi-steady-state analysis. As each trace was observed to reach a desired discrete temperature through the thermal camera, the voltage and current of the power supply were recorded to derive the power input. Each power input was then normalized by the volume of the conductive traces as measured by a confocal microscope and assuming a rectangular cross section (Figure 3f). The linear relationship established here demonstrates that the transition temperature of this specific LCE formulation can be achieved through this stretchable circuitry. For future studies that seek to adapt this technology, the relationship will also allow them to determine what power input is required for the LCE with different transition temperatures, which can accelerate the proliferation of this technique to a variety of heat-responsive materials. It is recommended to use power inputs that drive the temperature of the underlying material to just above the desired *T*_NI_ for steady-state control applications. Some Joule heated actuators with varying condutive trace designs are demonstrated in Figure S7.

### Adherence of Conductive Ink to LCE

A 90° peel test was conducted to determine if the ink would delaminate from the LCE surface under an applied load. The results of this experiment are shown in Figure S8. While force data could be collected for this experiment, all of the samples failed in cohesion before they failed in adhesion under the loading conditions. Qualitatively, this test shows that the ink can bind well to the LCE surface. No delamination at the interface between the LCE substrate and printed ink was observed in any of the structures that were implemented.

### Contribution of the Conductive Ink to Flexural Rigidity

To give a qualitative demonstration of the effect of the conductive ink on the mechanical properties of the actuator, Figure S9 is introduced. In this figure, two bending actuators are introduced with different Joule heating geometries (Figure S9a,b). These two actuators were designed to test how the flexural rigidity of the added conductive ink may affect the performance of each actuator—the actuator in Figure S8a should have a higher flexural rigidity than the actuator in Figure S9b because more of the ink is concentrated on the axis of contraction in the former. Despite this, each actuator was able to achieve a similar level of curvature (Figure S9c,d) through Joule heating with a 15 V power supply. The difference in actuation time is attributed to the higher resistance of the first pattern, which reduces the overall power being supplied to the system. These actuators suggest that for thicker, or higher aspect ratio, LCE structures, the contribution of the conductive ink to the performance of the actuator is minimal.

### Demonstrations

**Figure 4:**
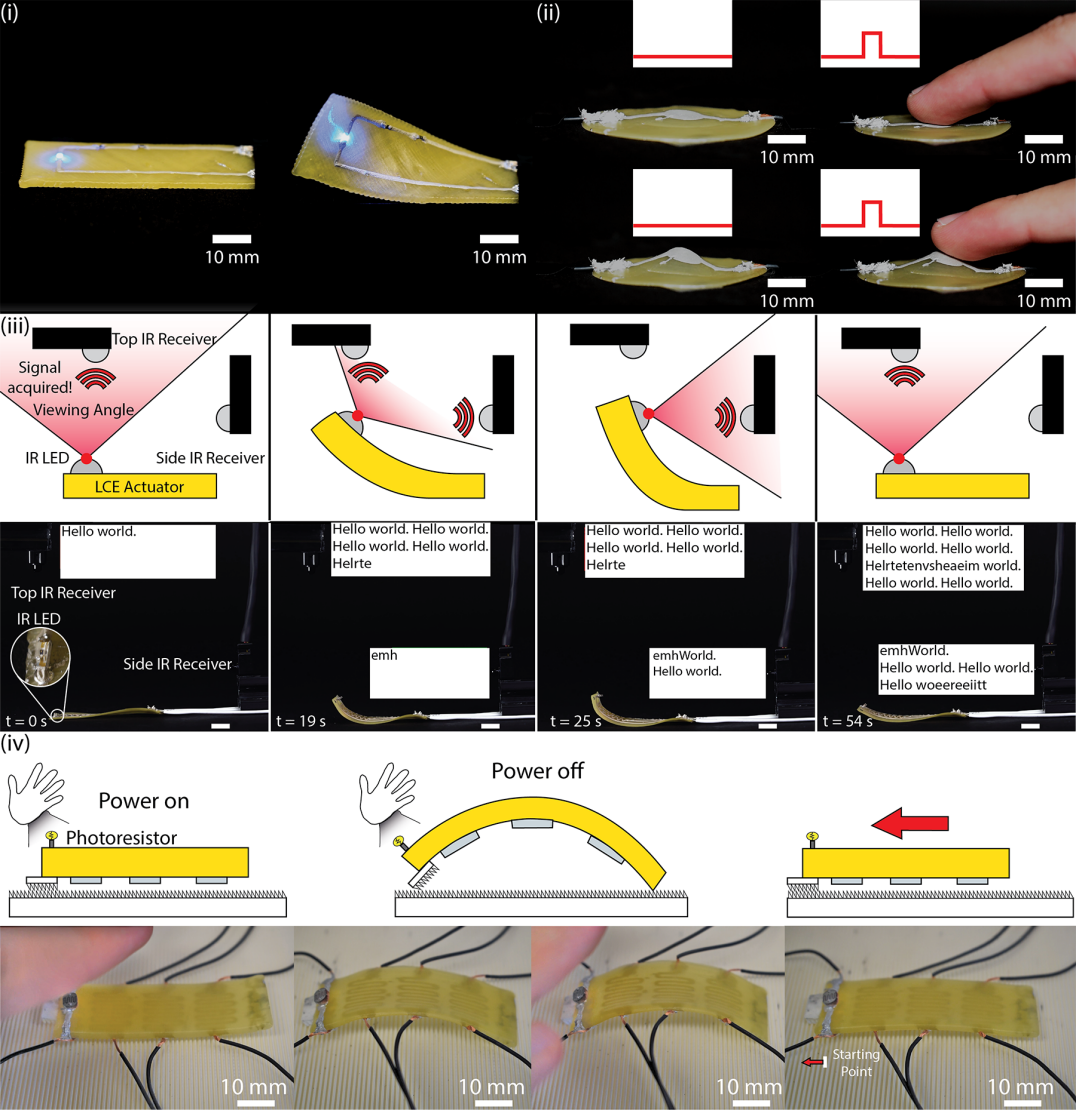
Shape reconfigurable devices using LCE and conductive ink. (i) A twisting actuator with SMD resistors and LEDs that can operate through a 90° twist. (ii) An LCE circle that pops up into a cone with a capacitive touch sensor. (iii) An LCE bending actuator with an infrared LED that blinks out a Morse code signal to two receivers. As the actuator bends, the viewing angle of the LED changes, which in turn changes the direction of the signal. (iv) An LCE crawler with an on/off photoresistor. Using 3D printed feet, the crawler can store energy as it is heated and then pull itself forward as it cools.

To demonstrate the versatility of this manufacturing approach, several LCE actuators with different behaviors were manufactured and conductive traces were printed on top of them with a variety of purposes (Figures 4 and S10). In the first example, a twisting LCE actuator with surface mount resistors and LEDs is demonstrated. Despite undergoing a 90° rotation, the LED continues to stay as bright as it was when at rest (Movie S3). In the second example, an LCE actuator that pops up into a cone provides capacitive sensing. By placing conductive ink in a circular pattern, a conductive pad for sensing was constructed in the center of the circular substrate. Even as the LCE strains during actuation, the capacitive sensor still works with a similar signal-to-noise ratio (Movie S4).

In addition to providing circuitry for SMDs and sensors, the conductive ink can also double as Joule heating traces as previously mentioned. For the third example, an LCE bending actuator with an infrared (IR) LED (Movie S5) is heated through a power supply (not shown). The IR LED attached to the bending end is connected to an Arduino microcontroller that controls when it is powered. The IR LED is powered by a conductive ink trace that is electrically separate from the heating trace. By augmenting the device with an IR LED, a reconfigurable antenna can be achieved, broadcasting the message “Hello World”. When powered off, the device is resting with the IR LED facing toward the ceiling. While both IR receivers in frame are powered, only the one positioned directly above the device is receiving data at *t* = 0 s. As the power supply is turned on, heat begins to accumulate on the device, causing bending. At *t* = 19 s, the communication signal is not fully captured by either receiver, distorting the transmission received by each signal. This is because the viewing angle for the IR LED is wide enough that an intermediate state where both IR receivers obtain the data signal can be achieved. During this intermediate stage, some of the LED blinks are registered to the top receiver, and others are registered to the side receiver, meaning each one receives and attempts to parse only a part of the whole signal. As the bending continues (*t* = 25 s), the IR receiver on the right captures the entirety of the signal, and information is no longer coming into the top receiver. The actuator is held there for a couple of seconds before the power to the Joule heating trace is removed, causing the device to relax to its original flat state as heat leaves the system. As it reaches its initial state, the receiver on the right stops receiving information, and the top one begins to receive the information once more.

As an additional demonstration, an LCE soft robotic crawler with integrated photosensing capability was fabricated using this approach (Figure 4 and Movie S6). Conductive ink is patterned on both sides of the actuator, with the Joule heating circuitry on the underside and the electronics on the upper side. The full circuit for controlling the actuator is given in Figure S11. The actuator can move by transitioning to its curved configuration and then using the attached feet to drag itself forward. A simple on/off control scheme for supplying heat to the actuator is developed in which crawling motion is triggered by light detected with an integrated photoresistor. The actuator starts at rest with no power being drawn to the heaters. The user then places their hand over the photoresistor to dim the light. This activates a threshold check in the microcontroller to allow the heaters to draw power. As the actuator heats, it curls, storing energy that will push the foot forward along the textured ground. Using the same photoresistor, the power can be turned off. As the actuator relaxes, it drives itself forward and uses the attached foot to secure its progress.

## Discussion

The major focus of this work was on introducing the manufacturing techniques used to create composite LCE actuators with integrated stretchable circuitry. To achieve these goals, the digital fabrication process had to be developed in order to create multimaterial structures. Once this was accomplished, we examined the properties of the printed materials to ensure that they retained their functionality.

One key insight into developing the printing method is that it is necessary to add an extra layer of LCE that is not UV cured in order to ensure a smooth surface finish on prints that are larger than a single layer. This is especially important if printing conductive ink on the LCE substrate. Further iteration of the printing parameters as well as customized G-code that prioritizes continuity in the toolpath could partially solve this issue—many of the defects that come from printing are formed during layer changes due to excessive nonworking moves, when the nozzle leaks a small amount of material that can end up in the final structure. However, adding the extra layer of LCE provides for a more generalized approach that allows researchers to work with readily available slicing software. This process is also useful for creating high aspect ratio LCE structures—while most of the previous works use either LCE thin films or only a couple of layers of LCE, the structures featured in this work are approximately 1.25 mm in thickness. Future work on this subject could lead to development of reconfigurable LCE antennas, an application for which surface roughness and waviness become important.^45^

To the best of our knowledge, the devices demonstrated in Figure 4 and Movies S3–S6 are some of the first examples in which coprinted LCE shape memory, integrated soft electronics, and SMDs are combined to operate a shape reconfigurable system. While some previous works have used through-hole LEDs,^28^ the pick-and-place approach of this method allows for the placement of SMDs, which can provide more functionality than what is shown in previous studies. The IR LED and photoresistors used in these demonstrations are simple components, but this approach can be leveraged for other ICs as well. A future aspect of this work that could be improved is through the implementation of feedback control to reach desired curvatures or actuation states. As seen in Figure 4 as well as in Movies S5 and S6, the Joule heating of the actuator is controlled only by an on/off switch and thus must be closely monitored to prevent the material from overheating or deviating from the desired position. By including onboard proprioception and feedback control, the antenna can be directed and held at specified positions, improving its communication abilities.

The main contribution of this work focused on the integration of a novel conductive ink with LCE, but some further context on why this novelty is important should be addressed. In similar works published recently using resistive circuitry,^18,46^ the resistance of a silver wire element greatly increases even with the moderate strains that LCEs can develop (30%). In contrast, the conductive ink used in this study has been shown to have a very low electromechanical coupling.^38,39^ In order to drive Joule heating and power to surface mounted devices, it is important for the electromechanical coupling to be as low as possible for stable operation—if the resistance of the conductive trace changes, then the power consumed over the circuitry may change, which will impact the performance of SMDs and Joule heating circuits. While controllers could be designed to mitigate the impact of these resistance changes, this would still not address the fact that inks containing rigid fillers are more likely to experience failure after hundreds of cycles when compared to the conductive ink used here.

The approach outlined in this work facilitates the exploration of other topics of interest in the field of LCE actuators. Feedback control and onboard proprioception using soft conductive ink circuitry would allow for specified curvatures and configurations.^47^ Localized Joule heating could provide direct control over portions of the LCE, which could be useful in applications where sequential folding is important, such as origami. Combining all these topics together, there is potential to explore the development of an LCE actuator with both a reconfigurable structure and reconfigurable circuitry. For example, consider an LCE hinge connected to two substrates patterned with conductive ink such that the circuits are electrically separate. By actuating the hinge, the substrates could be brought together so that the conductive ink now forms a complete circuit, executing other computation or machinery once activated. In general, this approach allows for the inclusion of complex circuit patterns while not compromising the soft nature of the actuator; by patterning the ink on both sides of the surface, soft, reconfigurable printed circuit boards can be designed, including with vias if carefully designed. Overall, this approach can spawn a variety of different actuators and devices with advanced functionality that will empower researchers that use LCE to start using the material for more advanced applications.

Deformation cannot damage the rigid devices themselves but can damage their connection to the surface of the LCE. In this work, conductive ink or a silver conductive epoxy was applied to the actuators by hand to provide electrical and mechanical connections between the LCE and SMDs. These items were chosen for convenience as they were already readily available in the lab, but there is no reason that another type of conductive material could not be used to make these connections provided that (1) the placement of the SMDs with respect to the deformation has been taken into account during design and (2) the sintering or final formation of the connection does not require heat. It is better to place the rigid components on parts of the LCE surface where there are no Joule heating traces because the underlying LCE will not contract. On an actively contracting portion of the surface, the large deformations could lead to failure of the SMD connections due to delamination. For example, the end of the bending actuator that has the IR LED (Figure 4) is positioned away from the heating coils so that there is a smaller likelihood of delamination from the LCE surface. After geometrical and practical considerations, the next consideration is that of the interconnect material. The conductive ink provides a stretchable interconnect for SMDs, but this bond was unsuitable for connecting thicker wire elements to the LCE, as the wires would tend to fall out of position when the ink was drying. For these interconnects, a silver conductive epoxy was applied to provide a stronger, more rigid anchoring point. This conductive silver epoxy is placed away from the regions of highest deformation to ensure that they do not impact the behavior of the composite device. Other conductive materials other than the ones reported here can be substituted so long as they do not require heat to sinter because heat will trigger the deformation of the underlying LCE and lead to a poor connection between the SMDs and the LCE surface. For this reason, something like a typical thermal soldering method cannot be used.

There are areas of improvement that could be considered for future work. In order to create finer and higher resolution conductive traces, the concentration of toluene in the conductive ink could be further tuned to reduce the effect of spreading due to gravity. While the conductive ink tends to be difficult to smear by hand, it can still be damaged by sharp edges and abrasive surfaces. However, the ink works well even without encapsulation and is resistant to smearing by hand with a glove, which cannot be said of implementations using pure liquid metal. This is due to the presence of the silver particles in the ink, which provide an anchoring point for EGaIn droplets.^38^ Additionally, even with a slight amount of liquid metal leakage, the conductive composite can still function for several cycles due to the presence of the silver flakes. That said, encapsulation could help to prevent these issues, and further studies into this should be explored. Additionally, further characterizations on the interface between the conductive ink and the LCE surface should be conducted to understand the factors leading to the strong adherence to the LCE and low contribution to the overall structure’s flexural rigidity. Finally, the integration of advanced cooling techniques with semiconductor technology could be leveraged to improve the actuation performance of the composite structure.^48^

## Conclusion

A new digital fabrication methodology utilizing multimaterial printing of LCE and conductive LM inks is proposed and leveraged to design actuators powered by Joule heating and augmented with surface mount electronics. A smooth surface finish was required to ensure that the conductive ink could be patterned evenly and provide uniform heating over the desired area. To achieve this, the printing parameters were empirically tuned, and an additional layer of LCE without the UV light turned on during printing was added to each actuator. Conductive ink traces could be patterned reliably on LCE surfaces for 100+ traces with a 99.1% yield. Additionally, the conductive ink can function without a large change in resistance at the elevated temperatures required to actuate LCE; these desired temperatures can be achieved by deploying the conductive ink as Joule heating traces which follow a linear relationship with input power. Finally, several reconfigurable actuators, including a reconfigurable IR communication device and a crawler, were developed using this approach. In the future, this method can be used to create larger LCE actuators and develop intricate electrical behavior through the bending and folding of the structure, such as creating an electrical connection where one did not exist in the rest state. More broadly, this approach could have a potential impact in applications ranging from soft robotics to electrically powered origami structures that require tight integration of stimuli-responsive shape memory materials and embedded soft electronics.

## Experimental Section

### LCE Formulation

The liquid crystal monomer 1,4-bis[4-(3-acryloyloxypropyloxy)benzoyloxy]-2-methylbenzene (RM257, Wilshire Technologies) was mixed in a 1.1:1 molar ratio with *n*-butylamine (nBA, Sigma-Aldrich) along with 4 wt % of a photoinitiator, Omnirad-369 (2-benzyl-2-(dimethylamino)-1-[4-(morpholinyl)phenyl)]-1-butanone).^17^ The materials were placed in an oil bath held at 75 °C during mixing and oligomerization. The solution was mixed for 15 min at 500 rpm using a magnetic stirrer before being left to sit in the oil bath for 1 h to partially oligomerize.

### EGaIn-Ag-SIS Conductive Ink Formulation

To create the LM alloy, gallium and indium (Rotometals) were mixed in a 75:25% weight ratio on a hot plate at 200 °C until both metals were liquefied. Styrene–isoprene–styrene with 14 wt % styrene (SIS, Sigma-Aldrich) was mixed with toluene (Sigma-Aldrich) in a 15:85% weight ratio on a hot plate held at 100 °C to create an aqueous block copolymer solution. This solution was mixed with silver flakes (SF94, Ames-Goldsmith) and EGaIn in a ratio of 1 g of SIS solution for every 1.24 g of SF94 and every 3 g of EGaIn.^38^ The solution was mixed by hand in between the addition of each conductive filler to ensure that AgIn_2_ deposits did not form too early and clog the printing needles. Once the liquid metal was added, the materials were hand mixed for 1 min to break down the large drops of bulk EGaIn. The vial of ink was then loaded into a planetary THINKY AR-100 mixer and mixed for 3 min at 2000 rpm.

### LCE Fabrication

After partial oligomerization, the LCE ink is loaded into a 10 cm^3^ stainless steel reservoir and loaded into the KRA heated extruder on a Hyrel System 30M printer (Hyrel3D). The extruder is allowed to reach thermal equilibrium at 65 °C for 15 min before starting any prints. The LCE ink is printed at 65 °C at a speed of 5 mm s^–1^ using a 22G metal nozzle (inner diameter 0.453 mm, McMaster-Carr). Other printing parameters are in the Supporting Information (Table S1). The ink is printed onto a transparency film (3M). To lock the mesogens into place during printing, a UV array with 365 nm LEDs is run at a 20% duty cycle during printing (Movie S1). To print a final smooth layer, the UV light is turned off. After printing, the sample is transferred to a UVP CL-1000 cross-linker for 10 h to fully cure.

### EGaIn-Ag-SIS Ink Printing on LCE

Once the LCE sample is cured, the conductive ink is printed onto the top surface of it. The conductive ink is loaded into a 10 mL syringe and then placed inside an SDS-10 extruder (Hyrel3D). Next, the LCE sample is secured to the printing bed and calibrated to match the printing profile of the conductive traces. Other relevant printing parameters are documented in Table S1. The traces are printed for using a 27G needle (inner diameter 0.210 mm, McMaster-Carr) (Movie S2). After printing, the sample is transferred to a fume hood to allow the toluene to evaporate out of the ink for 1 day.

### Pick and Place Components and Leads

Once the ink has cured, the SMD components and 26 AWG flexible wires are pick and placed onto the conductive traces. Additional conductive ink is used to secure SMD components while a conductive silver epoxy (8331D, MG Chemicals) is applied to the leads to secure them to the structures. The epoxy is mixed using equal parts of the resin and hardener. The device is left to sit for 1 day to ensure that the epoxy hardens and that the solvent evaporates from the ink.

### Characterization of Nematic-to-Isotropic Transition Temperature

The transition temperature *T*_NI_ of the LCE ink formulation was captured using a differential scanning calorimeter. The sample was allowed to reach thermal equilibrium at −50 °C before being ramped to 150 °C at 10 °C/min and then back down to −50 °C. This occurs for three cycles, during which the heat flow data are collected. The data displayed are from the second heating/cooling cycle.

### Rheological Characterization

The frequency sweeps and viscosity tests were conducted using a DHR-2 stress-controlled rheometer (TA Instruments) with a 40 mm diameter plate probe and a 1 mm gap. For the LCE ink, the sample was held at 65 °C for each test to simulate printing conditions. Each sample was tested using a logarithmic sweep from 10^–1^ to 10^2^ rad s^–1^ for angular frequency and 10^–1^ to 10^2^ s^–1^ for shear rate.

### Confocal Microscopy for Surface Roughness and Imaging

The roughness and waviness of the LCE surface are captured on a confocal microscope (Zeiss LSM 900) with the objective lens set to 10× with a numerical aperture of 0.4. After focusing the sample, a topography procedure installed in the system is used to extract the 3D reconstruction of the surface and the profiles along specified paths. Any nonmeasured points are filled in using a smooth approximation from neighboring values. This microscope was also used to capture images of the conductive ink structures using a 5× objective lens.

### Polarized Optical Microscopy of LCE Surfaces

Polarized optical microscopy was performed using a Zeiss Axio Imager Z2 Vario with both white light and reflected polarized light on two different samples. Each sample is a twisting actuator with two layers (50 mm × 10 mm × 0.5 mm), where the 3D printed traces are printed at antiparallel 45° angles. However, one sample’s top layer was printed without using UV light during the process. The polarizer and analyzer were set to be orthogonal to one another, and a 200 ms exposure time was used. Auto white balance and best fits were applied to all captured images.

### Contractile Tests

Each linear actuator was a 3D printed sample of rectangular dimensions 10 mm × 50 mm, with varying numbers of layers (1 layer, 2 layer, and 3 layer structures, with each layer being 0.25 mm in height). However, some of the samples are printed with the extra planarization layer, and some are printed without it. Three samples (*n* = 3) were used in each group. The contraction ratios were measured by capturing videos of each actuator while being exposed to a heat gun for 10 s at the highest temperature setting (SEEKONE, 650 °C). Starting and ending configurations were selected from the videos and analyzed in ImageJ along with a known distance to measure the length of each sample before and after actuation (*l*_i_ and *l*_f_). The contraction ratio is calculated as . To compare the samples to the assumed power level of 0.01, a student’s two sample *t* test while assuming equal variance for a single tailed test was calculated in Microsoft Excel.

### EGaIn-Ag-SIS Ink Repeatability

A total of 36 groups of 3 conductive traces were printed on the same LCE substrate. Each line has a length, , of 5 mm. The traces were designed to be continuous to reduce the chance of a large bubble of material forming due to nonworking moves from the printer. The width, *w*, of each traces was measured using a confocal microscope with a 10× objective lens. The height, *h*, of each trace was measured by sweeping the focus of the microscope from the bottom of the sample to the top of the sample. Measurements were conducted at the midpoint of each line. The resistance, *R*, of each trace was measured using a digital multimeter (Agilent) set to a 4-wire resistance measurement. Small pieces of wire were used to probe the material—the resistance of these wire elements was recorded and subtracted from the recorded resistances. The conductivities were calculated using Pouillet’s law and the other measured parameters:
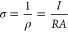
1where σ is the conductivity, ρ is the resistivity, and *A* = *wh* represents the effective cross-sectional area of the trace (assuming a rectangular cross section).

### IR Imaging

IR images and videos are captured using a FLIR A35 thermal camera at 60 frames^-1^. A rainbow palette with a linear, fixed temperature scale is used to better contrast the heated LCE from the surroundings.

### Thermal Analysis Measurements

For the first experiment, a benchtop multimeter (Agilent 34401A) was set to capture resistance data using a 4-wire resistance measurement. Each sample was heated using a hot plate, and the resistances were manually recorded at 5 °C increments. For the second experiment, each line was Joule heated using a benchtop power supply. The voltage, current, and power were recorded manually from the power supply screen at 5 °C increments.

To normalize the power input, the trace cross-sectional area (height and width) were measured using the aforementioned confocal microscope. The length of each trace was measured using a digital caliper. The volume was assumed to be a rectangular prism, with *V* = *lwh*.

### Peel Test

A modified 90° peel test was used to test the adhesion strength of the ink to the LCE. The ink is printed in a rectangular pattern (10 mm × 40 mm) directly onto an LCE strip measuring 20 mm × 60 mm. Masking tape is placed on half of the desired area to ensure that this section could be peeled back prior to the test. The sample is loaded on an Instron Universal Testing Machine with a custom grip to hold the sample down. A tensile method with a rate of 10 mm min^–1^ was used.

### Reconfigurable IR Communication Demonstration Components

IR receivers were purchased and used as is from Adafruit. An SMD IR LED operating at 940 nm (QT-Brightek) was powered by an Arduino Uno sending out a repeating signal. A separate Arduino Uno was used to capture the incoming signal on both receivers and display it using the tkinter library in Python. The references to the code are included in the Supporting Information. A power supply is held at 15 V to power the Joule heating circuit.

### LCE Crawler Demonstration

Through hole photoresistors were purchased from Adafruit and cut to approach the form factor of an SMD. Conductive ink was printed on both sides of the actuator to create the heating circuits and power lines for the photoresistor. The board and feet were manufactured using PLA on an Ultimaker S5 3D printer. The foot was attached to the underside of the actuator using Sil-Poxy (Smooth-On). The references to the code and the circuit wiring are included in the Supporting Information (Figure S6). A power supply is held at 5 V to power the Joule heating circuit.

## Supporting Information Available

Additional experimental details, results/figures, photographs of printing and experimental setups and actuators (PDF)
Movie S1 (MP4)
Movie S2 (MP4)
Movie S3 (MP4)
Movie S4 (MP4)
Movie S5 (MP4)
Movie S6 (MP4)
