# Supplementary material for: Multimaterial Printing of Liquid Crystal Elastomers with Integrated Stretchable Electronics
Source: ACS Appl Mater Interfaces. 2023 May 10;15(20):24777–87. doi: 10.1021/acsami.2c23028 (PMC10214374; doi:10.1021/acsami.2c23028)
Supplement: Supplementary file 1 — am2c23028_si_001.pdf [file am2c23028_si_001.pdf]

Supporting Information

Multi-Material Printing of Liquid Crystal Elastomers with Integrated Stretchable Electronics

Michael R. Vinciguerra[*,†], *Dinesh K. Patel*[‡], *Wuzhou Zu*[+,†], *Mahmoud Tavakoli*[*,⁋], *Carmel Majidi*[*,†], *Lining Yao*[*,‡]

† Department of Mechanical Engineering, Carnegie Mellon University, 5000 Forbes Ave., Pittsburgh, PA 15213, USA

‡ Human Computer Interaction Institute, Carnegie Mellon University, 5000 Forbes Ave., Pittsburgh, PA 15213, USA

⁋ Institute of Systems and Robotics, Department of Electrical Engineering, University of Coimbra, Coimbra 3090-290, Portugal

+ Present Address: Department of Mechanical Engineering, Virginia Tech, Blacksburg, VA 24060, USA

E-mail: mvincigu@andrew.cmu.edu; mahmoud@isr.uc.pt; cmajidi@andrew.cmu.edu; liningy@andrew.cmu.edu

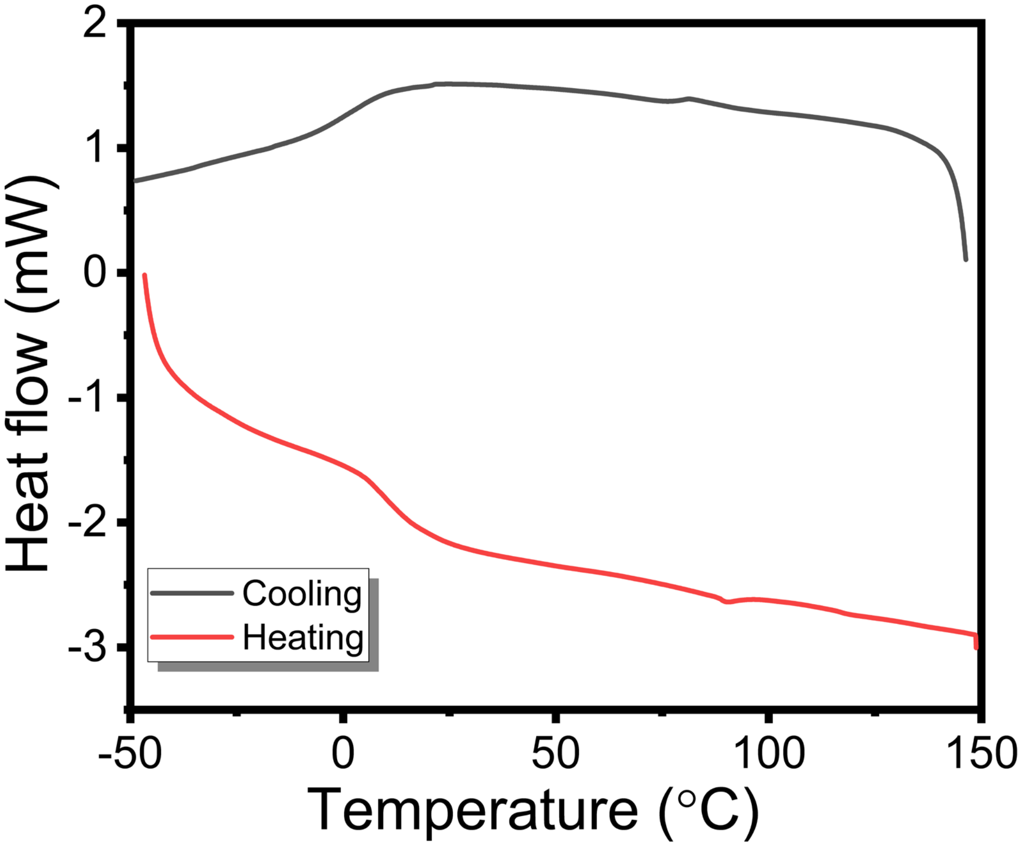

Figure S1. Differential Scanning Calorimetry. The nematic-to-isotropic transition peaks occur roughly around 90°C, suggesting that the conductive heaters need to reach this temperature to fully actuate the LCE.

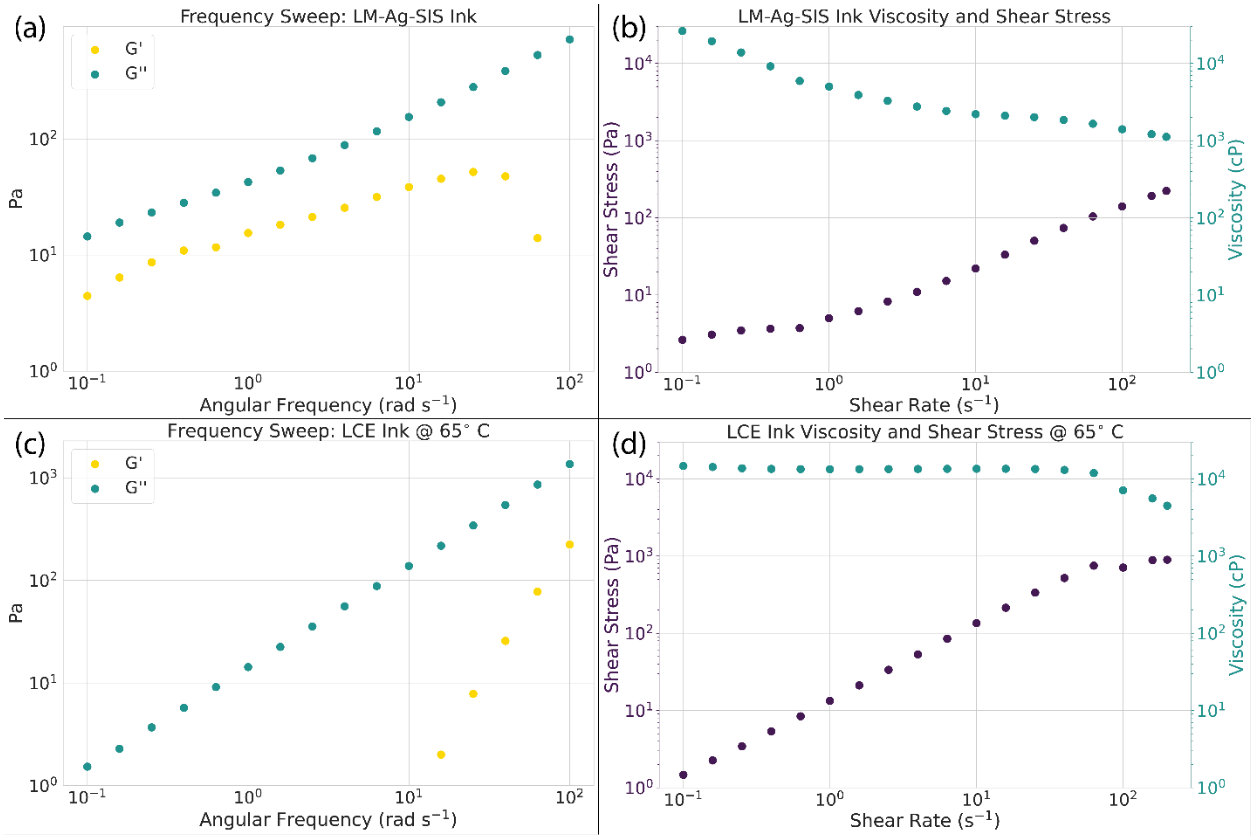

Figure S2: Rheology characterization of conductive and LCE inks. (a) Frequency sweep of LM-Ag-SIS Ink. (b) LM-Ag-SIS Ink shear stress and viscosity as a function of shear rate. SDS-10 extruder on Hyrel is capable of extruding materials up to 10^5 cP. (c) Frequency sweep of LCE ink at printing temperature of 65 degrees C. Data cannot be displayed for the storage modulus at small angular frequencies due to noise. (d) LCE Ink shear stress and viscosity as a function of shear rate at printing temperature of 65 degrees C. KRA extruder on Hyrel is capable of extruding materials up to 10^6 cP.

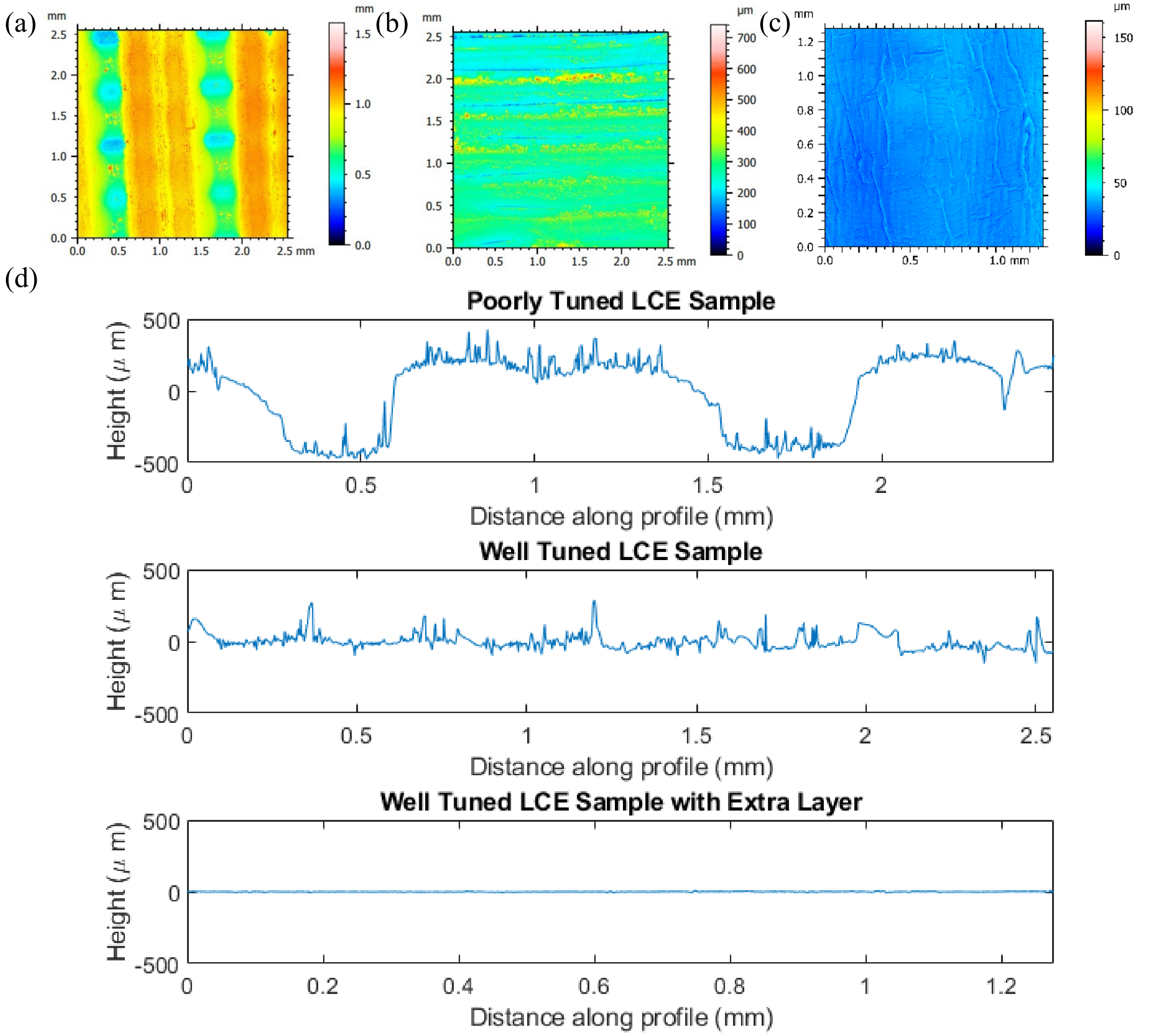

Figure S3. Confocal Analysis of LCE Surfaces. (a) An LCE sample without empirical tuning. There are large gaps in the structure that prohibit the printing of conductive traces. (b) An LCE sample printed with empirically tuned parameters. While there are no gaps, there are small defects that can make it difficult to print the conductive ink. (c) An LCE sample printed with empirically tuned parameters and an additional layer of LCE with the UV light turned off. The surface is relatively uniform and smooth. (d) Height plots along a single path for each sample from a-c. Without tuning, large gaps between single LCE fibers are observed. With tuning, there are no large gaps, but smaller defects can still occur. With the addition of the final layer of LCE without the UV light turned off, the maximum deviation in the surface height is <10μm.

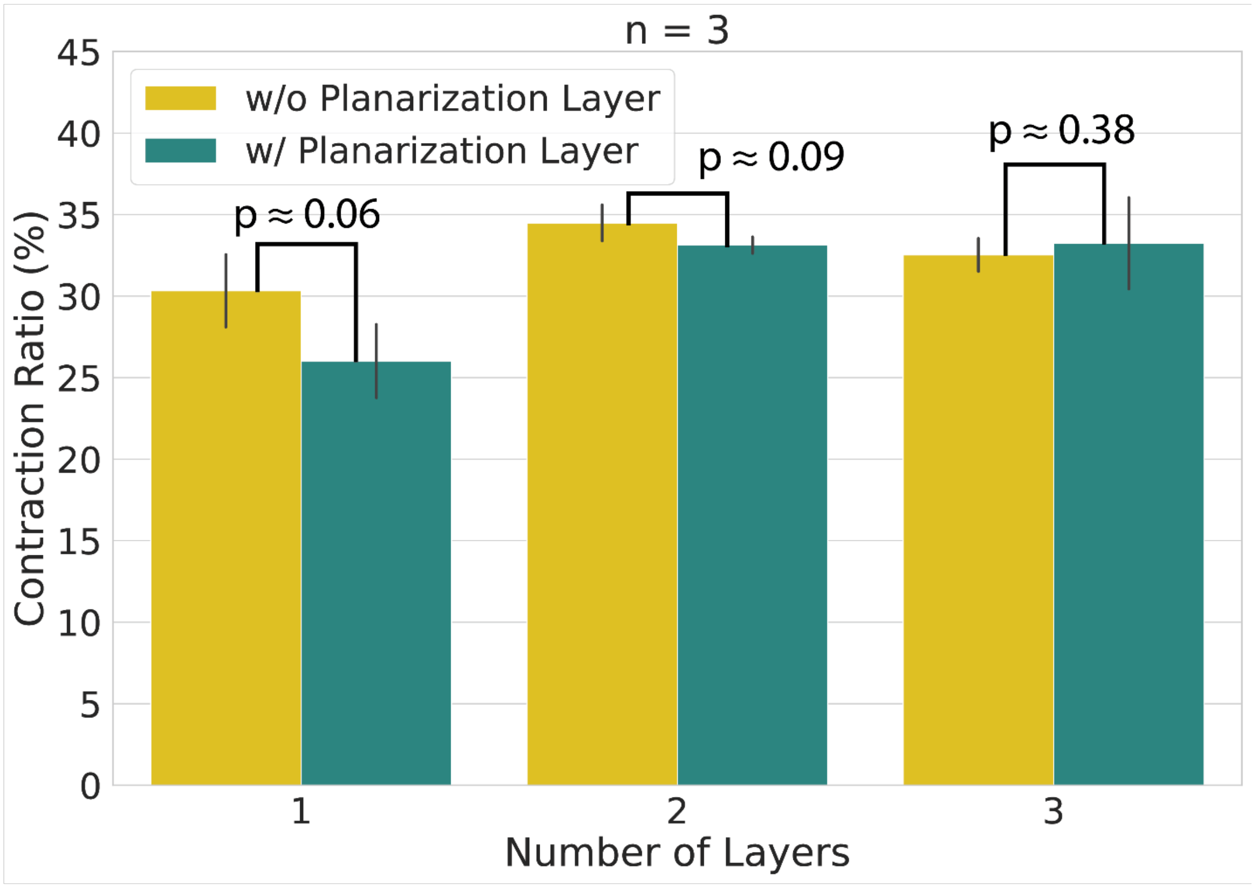

Figure S4: Averages of contraction tests of 3D printed LCE actuators with and without an extra planarization layer. The black lines indicate standard sample deviations. The number of layers corresponds to the number of layers with the UV light turned on. Each test uses 3 samples. Probability scores are calculated using a student's two sample T-test with equal variance. Scores indicate that there is no significant difference between the samples with the extra layer and those without it.

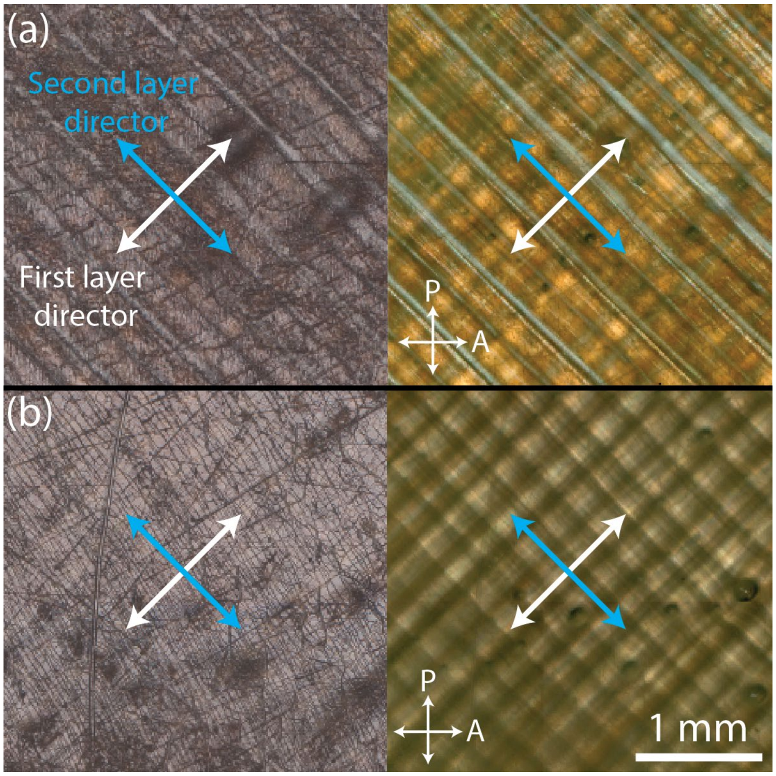

Figure S5: Polarized optical microscopy of twisting actuator. (a) A two-layer sample with the UV light turned on for both layers. Left - the sample under a brightfield. Right - the same sample under polarized light shows the birefringence of the printed LCE, allowing a viewer to see both layers. (b) A two-layer sample with the UV light turned off for the second layer. While the sample is dimmer under polarized light, the printed lines are still visible, suggesting that the sample is still mostly monodomain. All pictures use the same scale bar.

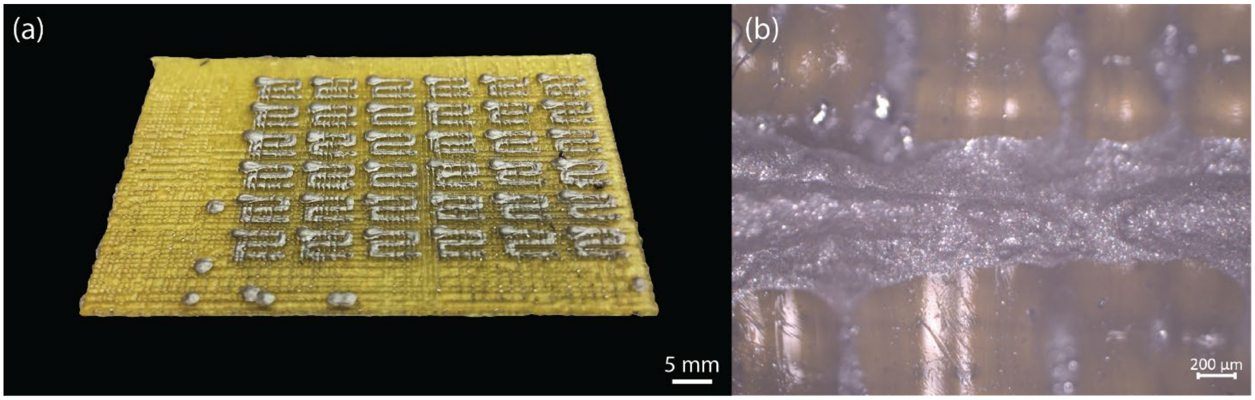

Figure S6: Repeatability prints on an LCE substrate without the addition of the planarization layer. (a) The surface of the LCE substrate with all prints shown. (b) A confocal image of a representative trace from the surface shown in (a). The gaps between LCE traces cause the conductive ink traces to be printed very poorly, rendering further experimentation difficult.

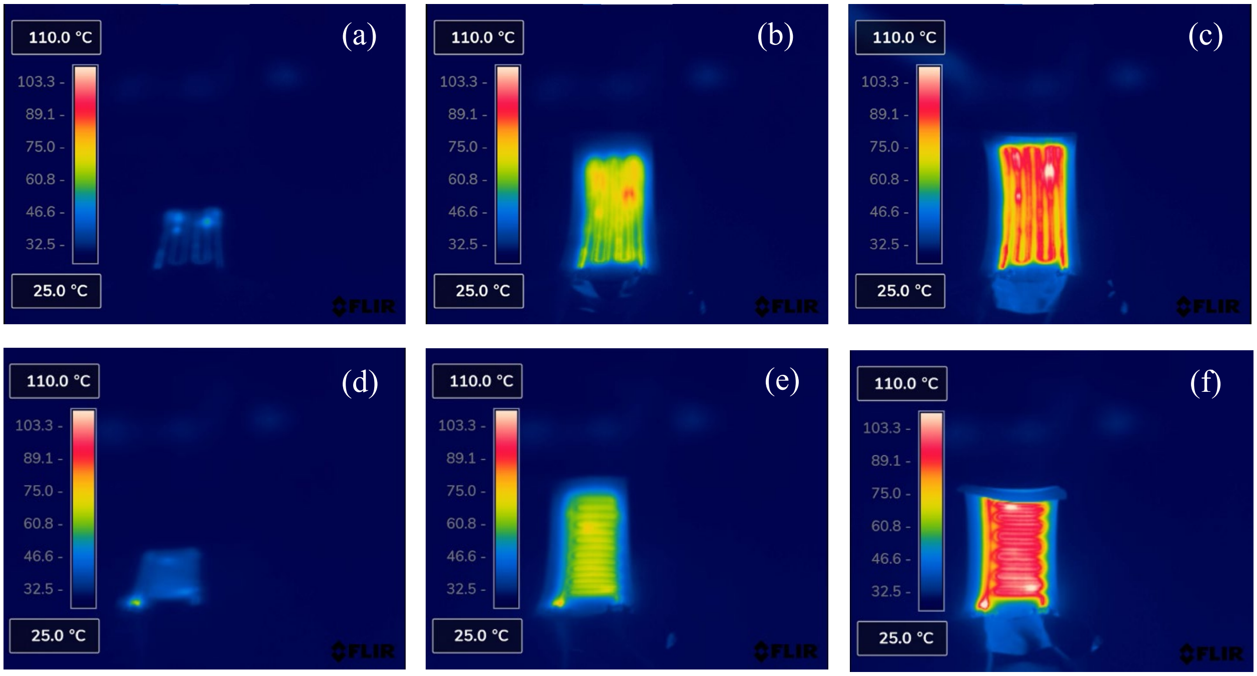

Figure S7. LCE Actuator Localized Joule Heating. Each actuator is powered using a voltage power supply held at 15V. (a-c) An LCE actuator with conductive ink traces aligned with the axis of bending at three different times. The outline of the traces contrasted against the LCE surface demonstrates the localized heating. (d-f) An LCE actuator with conductive ink traces aligned against the axis of bending at three different times. The heating is more uniform across the surface when compared to (a-c) due to the tighter packing of the conductive traces, leading to faster actuation (see Figure 3).

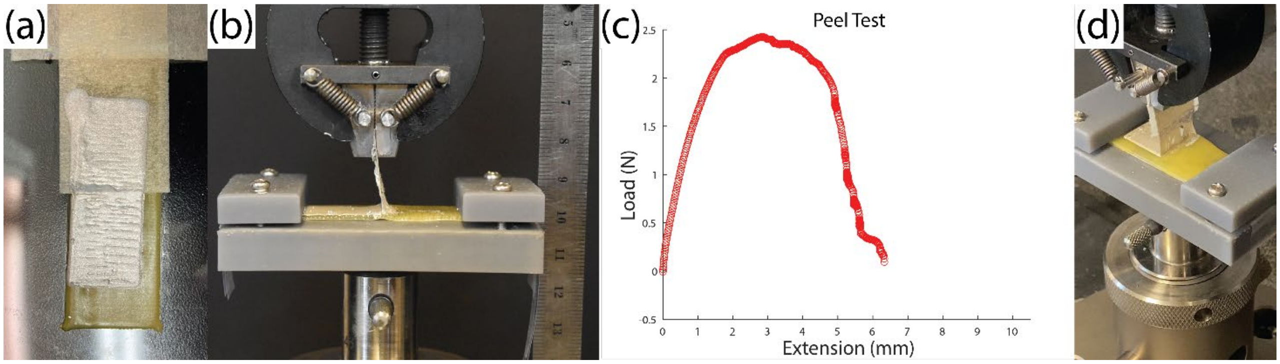

Figure S8. Peel test. (a) Sample of printed conductive ink on LCE substrate. The masking tape is placed prior to printing to ensure easy removal of one section. (b) Loading of the sample onto the Instron machine. The sample is tightened down with a custom stand. (c) Force data captured by the Instron. (d) Samples fail in cohesion rather than adhesion as evidenced by the holes forming in the ink, suggesting that the ink bonds well with the LCE.

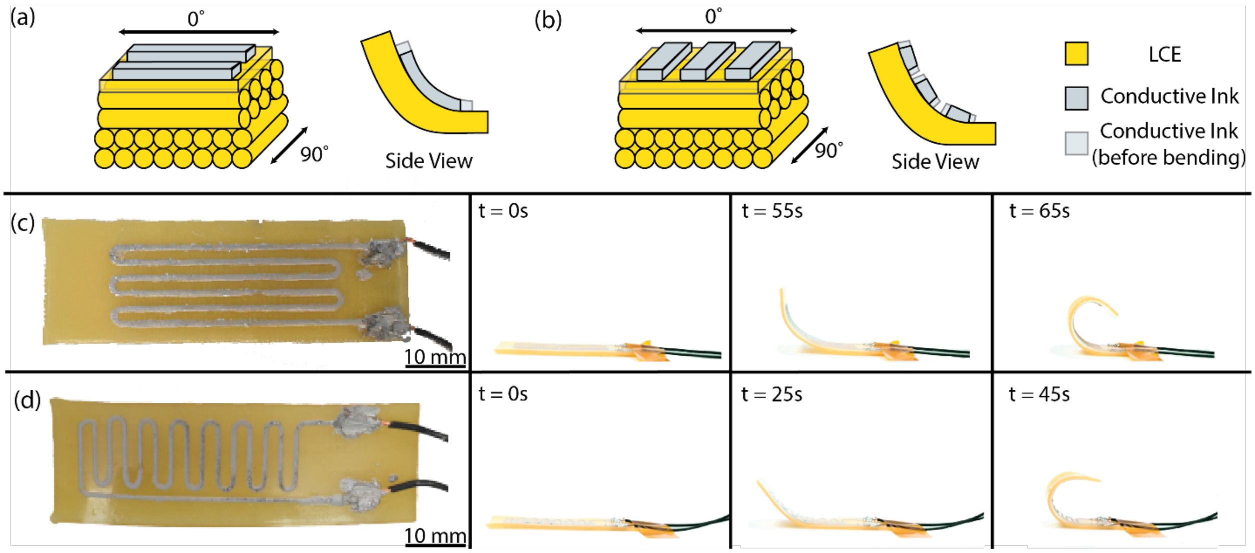

Figure S9: Thermal actuation of an LCE bending actuator with different conductive ink patterns. (a) The first pattern, which should theoretically increase the flexural rigidity of the actuator. (b) The second pattern, which should theoretically increase the flexural rigidity of the actuator less than the pattern shown in (a). (c) Thermal actuation of pattern one. (d) Thermal actuation of pattern two.

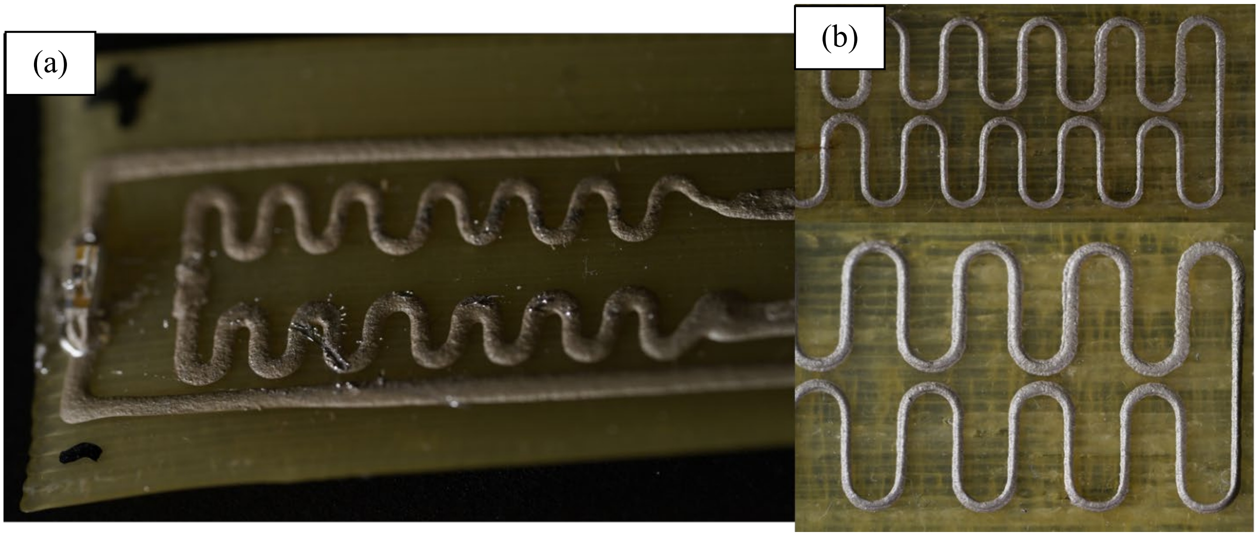

Figure S10. Examples of printed conductive traces. (a) A close-up of the actuator used in the demo showing the heating traces and power lines for the IR LED. (b) Close-ups of conductive traces on LCE substrates.

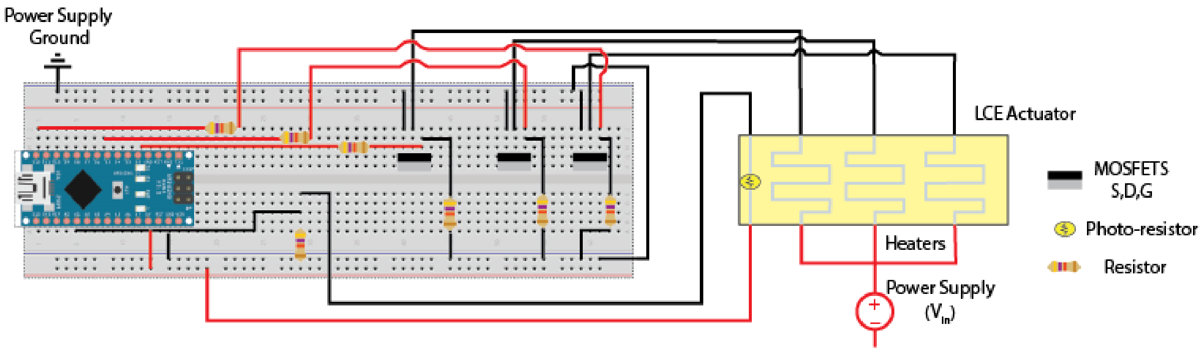

Figure S11. Full circuitry for the LCE crawler. Heaters (on the bottom of the crawler) are turned on and off using MOSFETS, whose gates are controlled by the digital pins of the Arduino Nano. Pull-up and pull-down resistors for the gates are all 1 kΩ and 10 kΩ, respectively. A power supply held at 5V is used to power the heaters. The photo-resistor (on the top of the crawler) is powered using the 5V pin from the Arduino and is put in series with a 4.7 kΩ resistor. Code for demos can be found at: https://github.com/softmachineslab/LCE_printing_demos

Ink	Extrusion Flow Rate Multiplier	Motor Speed (Pulses/µL)	Nozzle Speed (mm/s)	First Layer Width (mm)	Layer Width (mm)	Layer Height (mm)
LCE	0.60	1200	5	0.35	0.45	0.25
Ag-EGaIn-SIS	1.0	62.5	5	0.50	0.50	0.15

Table S1. Extrusion Flow Rate Parameters. A table summarizing the most relevant flow rate parameters for each of the DIW inks used in this work. The extrusion flow rate multiplier, motor speed, nozzle speed and trace cross-sectional area determines how much material each extruder attempts to output.

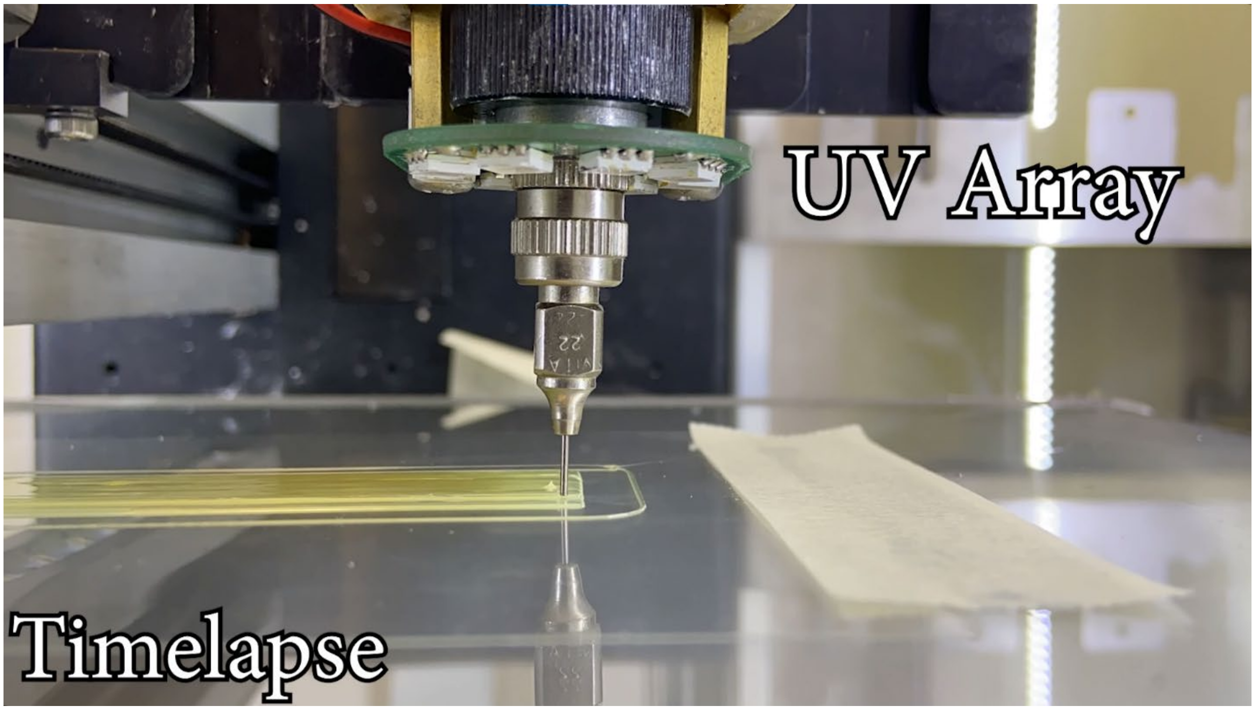

Movie S1. Timelapse of LCE Printing.

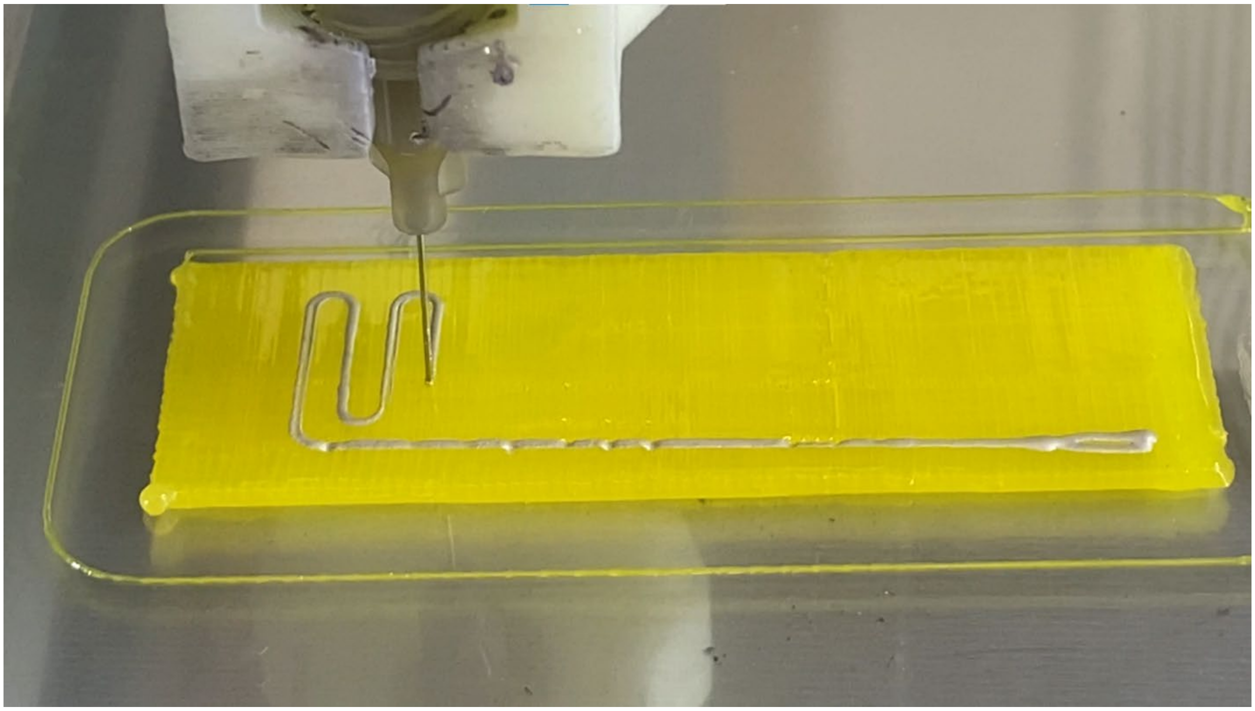

Movie S2. Printing the conductive ink on top of the LCE surface.

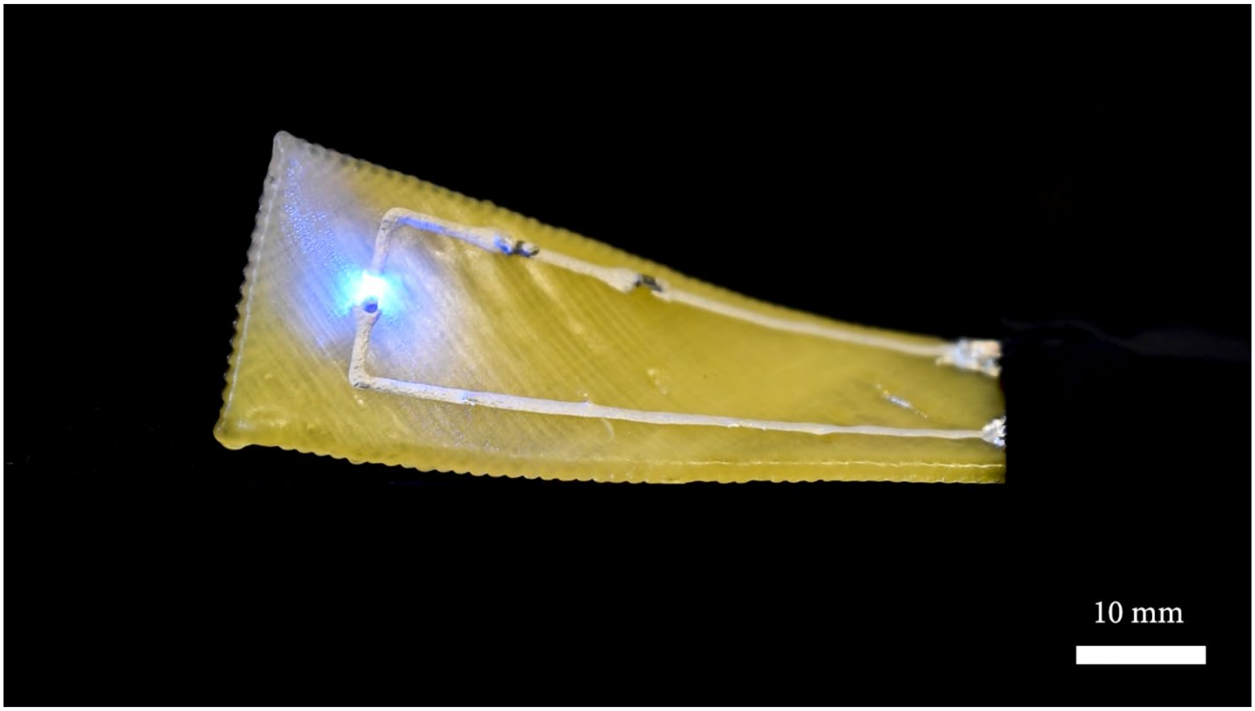

Movie S3. Twisting LCE actuator with SMD resistors and LED.

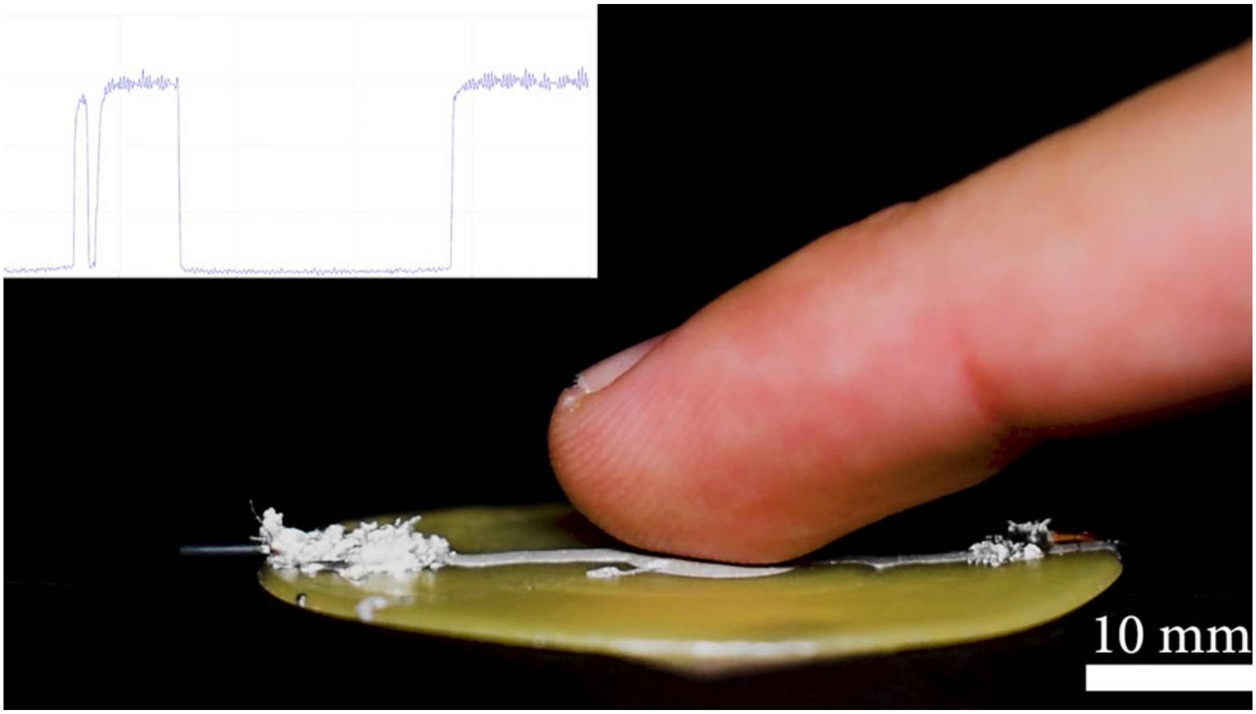

Movie S4. LCE Cone with capacitive touch sensor. Capacitive touch sensor is controlled using the Arduino Capacitive Sensor library.

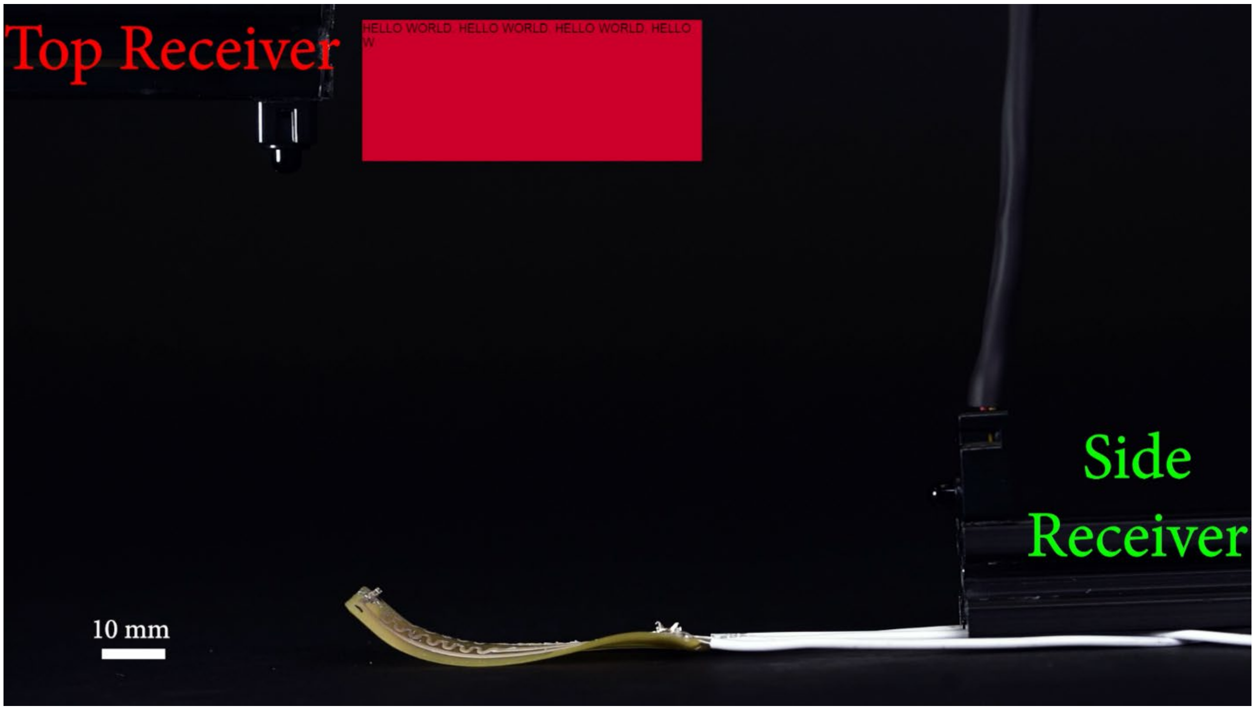

Movie S5. IR Communication Demonstration. Code to run the demonstration can be found at: https://github.com/softmachineslab/LCE_printing_demos

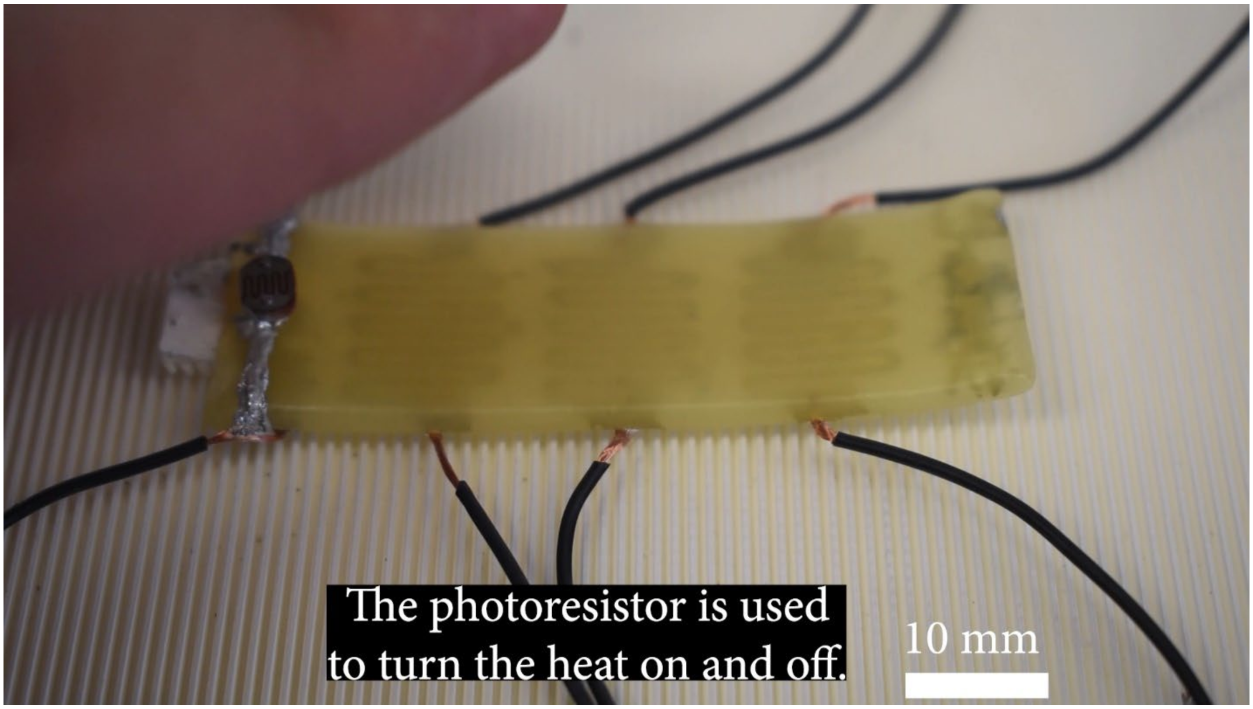

Movie S6. LCE Crawler Demonstration. Code to run the demonstration can be found at: https://github.com/softmachineslab/LCE_printing_demos
